# Relationships Between Gastrointestinal Parasite Infections and the Fecal Microbiome in Free-Ranging Western Lowland Gorillas

**DOI:** 10.3389/fmicb.2018.01202

**Published:** 2018-06-15

**Authors:** Klára Vlčková, Barbora Pafčo, Klára J. Petrželková, David Modrý, Angelique Todd, Carl J. Yeoman, Manolito Torralba, Brenda A. Wilson, Rebecca M. Stumpf, Bryan A. White, Karen E. Nelson, Steven R. Leigh, Andres Gomez

**Affiliations:** ^1^Department of Pathology and Parasitology, Faculty of Veterinary Medicine, University of Veterinary and Pharmaceutical Sciences Brno, Brno, Czechia; ^2^Institute of Vertebrate Biology, Czech Academy of Sciences, Brno, Czechia; ^3^Liberec Zoo, Liberec, Czechia; ^4^Institute of Parasitology, Biology Centre of the Czech Academy of Sciences, České Budějovice, Czechia; ^5^Central European Institute for Technology (CEITEC), University of Veterinary and Pharmaceutical Sciences Brno, Brno, Czechia; ^6^WWF, Dzanga Sangha Protected Areas, Bangui, Central African Republic; ^7^Department of Animal and Range Sciences, Montana State University, Bozeman, MT, United States; ^8^J. Craig Venter Institute, Rockville, MD, United States; ^9^Carl R. Woese Institute for Genomic Biology, University of Illinois at Urbana-Champaign, Urbana, IL, United States; ^10^Department of Microbiology, University of Illinois at Urbana-Champaign, Urbana, IL, United States; ^11^Department of Anthropology, University of Illinois at Urbana-Champaign, Urbana, IL, United States; ^12^J. Craig Venter Institute, La Jolla, CA, United States; ^13^Department of Anthropology, University of Colorado at Boulder, Boulder, CO, United States; ^14^Department of Animal Science, University of Minnesota, St Paul, MN, United States

**Keywords:** lowland gorilla, fecal microbiome, bacteria, parasite infection, *Entamoeba*, strongylid nematodes

## Abstract

Relationships between gastrointestinal parasites (GIPs) and the gastrointestinal microbiome (GIM) are widely discussed topics across mammalian species due to their possible impact on the host's health. GIPs may change the environment determining alterations in GIM composition. We evaluated the associations between GIP infections and fecal microbiome composition in two habituated and two unhabituated groups of wild western lowland gorillas (*Gorilla g. gorilla*) from Dzanga Sangha Protected Areas, Central African Republic. We examined 43 fecal samples for GIPs and quantified strongylid nematodes. We characterized fecal microbiome composition through 454 pyrosequencing of the V1-V3 region of the bacterial 16S rRNA gene. *Entamoeba* spp. infections were associated with significant differences in abundances of bacterial taxa that likely play important roles in nutrition and metabolism for the host, besides being characteristic members of the gorilla gut microbiome. We did not observe any relationships between relative abundances of several bacterial taxa and strongylid egg counts. Based on our findings, we suggest that there is a significant relationship between fecal microbiome and *Entamoeba* infection in wild gorillas. This study contributes to the overall knowledge about factors involved in modulating GIM communities in great apes.

## Introduction

The mammalian gastrointestinal microbiome (GIM) is essential in providing access to nutrients, regulating epithelial development, and shaping immunity (Berg, 1996; Isolauri et al., 2004; Eckburg et al., 2005; Round and Mazmanian, 2009; Consortium, 2012). Thus, determining the external and internal factors that impact the composition, metabolic activity and dynamics of the GIM are critical to understand host health (Mackie et al., 1999; Isolauri et al., 2004).

The impact of gastrointestinal parasite (GIP) infections on the composition and activity of the GIM has received increasing attention and several studies have been conducted in humans and animals (Berrilli et al., 2012; Cantacessi et al., 2014; Kreisinger et al., 2015; Morton et al., 2015; Šlapeta et al., 2015; Audebert et al., 2016; Zaiss and Harris, 2016). Great apes, as closest relatives to humans (Kumar et al., 2005), are useful models for microbiome studies, enabling microbiome patterns to be studied in the contexts of ecology, health, and evolution (Gomez et al., 2015, 2016b; Moeller et al., 2016; Vlčková et al., 2016). Previous studies on GIM of western lowland gorillas *Gorilla gorilla gorilla* showed that feeding behavior, geographical range and increased anthropogenic pressure may be important modulators of the gorilla GIM. Moreover, the GIMs of two gorilla species *G. gorilla and G. beringei* exhibit significantly different patterns, but the GIMs converge when hosts face similar dietary constraints, associated with low fruit availability in their habitats suggesting that in primates dietary constraints triggered during their adaptive radiation were potential factors behind the species-specific GIM patterns observed today (Ochman et al., 2010; Gomez et al., 2015, 2016b). Moreover, GIM communities of sympatric chimpanzees and lowland gorillas have converged in terms of community composition (Moeller et al., 2015). The overall composition of gorilla GIP corresponds to that of other non-ruminating herbivores with a dominance of entodiniomorphid ciliates (which are in fact commensals), strongylids and anoplocephalid tapeworms, especially in mountain gorillas (Kalousová, 2015). GIP infections of western lowland gorilla have been studied using traditional and advanced techniques at several sites, with most of the studies conducted in Dzanga Sangha Protected Areas (Landsoud-Soukate et al., 1995; Lilly et al., 2002; Masi et al., 2012; Sak et al., 2013; Hasegawa et al., 2014; Pafčo et al., 2017; Vlčková et al., 2018).

To our knowledge, there have been no studies focusing on the relationship between GIP infections and the GIM of free-ranging great apes yet. Human societies have always been challenged by infections by helminth and protozoan parasites and GIP have evolved with humans throughout history (Cox, 2002). It is hypothesized that a reduced exposure to potentially pathogenic organisms, including parasites, in developed countries may result in insufficient stimulation of the human immune system, leading to increased incidence of autoimmune, metabolic and allergic diseases in these populations (Reddy, 2010). Industrialized human populations are hypothesized to have lost a significant number of GIM signatures in comparison with non-human primates and traditional human groups such as hunter-gatherer populations (Moeller et al., 2014; Obregon-Tito et al., 2015; Gomez et al., 2016a). These losses are manifested through decreased abundances of taxa like *Treponema* and *Prevotella* and potentially linked to traditional dietary behaviors (i.e., more fibrous food) (Gomez et al., 2016a).

The metabolic products of the resident GIM may strongly interfere with the survival and the physiology of many parasites and consequently, influence the outcome of many parasitic infections. Conversely, GIPs, both protozoans and helminths, constantly excrete and secrete molecules (e.g., carbon dioxide, nitrogenous compounds, enzymes) that may change the gut environment, potentially leading to alterations in GIM composition (Berrilli et al., 2012). For instance, some helminths are able to significantly reduce inflammation during colitis (Elliott et al., 2007), potentially modulating alterations in colon epithelial barrier function (Su et al., 2011). Nematodes can alter the gut habitat, which may lead to alterations in bacterial composition and abundance (Walk et al., 2010). These changes can be either beneficial or harmful to the host. Wu et al. (2012) detected increases in the levels of *Campylobacter* in pigs infected with *Trichuris suis*, resulting in campylobacteriosis and decreases in *Fibrobacter* and *Ruminococcus*, with subsequent disruptions of fibrolytic activity. Various GIPs like *Blastocystis hominis, Giardia* spp., *Entamoeba histolytica, Dientamoeba fragilis*, and *Trichinella* spp. have been reported to contribute to the progress of irritable bowel syndrome in humans (Mohammadi et al., 2015).

Nematodes such as *T. suis* and their excretory/secretory products (multiple complex molecules including many (glyco)proteins, which may function as proteases or pore-forming proteins that could affect epithelial integrity) can suppress innate and adaptive pro-inflammatory immune responses (Whelan et al., 2012; Hiemstra et al., 2014), making them potentially suitable for the treatment of inflammatory bowel disease (Moreels and Pelckmans, 2005; Summers et al., 2005). Likewise, hookworm infection (e.g. *Necator americanus*) has been hypothesized to decrease gluten sensitivity and could serve as a potential treatment for celiac disease (Mohammadi et al., 2015). Moreover, amoebas from the genus *Entamoeba* significantly impact GIM composition and diversity in humans (Morton et al., 2015) and *Giardia intestinalis* causes alterations in the bacterial composition of the upper digestive tract (Tandon et al., 1977; Tomkins et al., 1978), with the GIM potentially influencing the clinical outcomes of giardiasis. The impact of GIPs on GIM composition and diversity have been recorded also in the case of *Giardia* infection in dogs (Šlapeta et al., 2015), helminths in wild rodents (Kreisinger et al., 2015), and *Heligmosomoides polygyrus* in mice (Walk et al., 2010). Nevertheless, the manner in which GIPs interact with resident gut microbes of primates remains poorly understood (Berrilli et al., 2012). It is assumed that a specific GIM predisposes an individual to parasite colonization, namely *Entamoeba* (Bracha et al., 1982; Galván-Moroyoqui et al., 2008). Burgess and Petri (2016) observed that GIM is a significant factor influencing the clinical presentation and outcome of *E. histolytica* infections. The GIM may significantly affect the virulence of the *Entamoeba* itself, its ability to colonize the gut, and the host's immune responses at baseline, and during amebiasis (Burgess and Petri, 2016; Ngobeni et al., 2017).

As a part of the long-term non-invasive monitoring of GIP infections and fecal microbiome/GIM of western lowland gorillas (*Gorilla g. gorilla*) in Dzanga Sangha Protected Areas (DSPA), Central African Republic (Sak et al., 2013; Hasegawa et al., 2014; Gomez et al., 2015, 2016b; Vlčková et al., 2016), we evaluate the associations between GIP infections and composition of fecal microbiome of free-ranging western lowland gorillas. We particularly focused on strongylid nematodes (*Necator, Oesophagostomum, Mammomonogamus*), which are highly prevalent in studied gorillas (Kalousová, 2013; Hasegawa et al., 2014; Kalousová et al., 2016; Červená et al., 2017). We included *Mammomonogamus* into the study, even though it is not a GIP. As the adults of this strongylid live in the respiratory tract of the host and only eggs are shed in the gastrointestinal tract, we did not expect the impact of *Mammomonogamus* on fecal microbiome. The following research questions were addressed: (i) Does the fecal microbial diversity differ between individuals positive and negative for particular GIP? (ii) Does the abundance of specific bacterial taxa differ between individuals positive and negative for particular GIP? (iii) Is there a relationship between fecal microbial diversity and surrogate measures of intensity of parasite strongylid infection (egg counts)? (iv) Is there a relationship between abundances of specific bacterial taxa and surrogate measures of intensity of parasite strongylid infection (egg counts)?

## Materials and methods

### Study site and sample collection

Fecal samples of western lowland gorillas (*G. g. gorilla*) were collected at two research sites: Mongambe (2°55′N, 16°23'E) and Bai Hokou (2°50′N, 16°28′E) in the Dzanga sector of the Dzanga-Ndoki National Park (part of the DSPA complex), Central African Republic (Figure S1). The samples were collected in 2011 during June through September, which corresponds to a rainy season with higher fruit availability. Samples from two habituated gorilla groups: H1 (9) and H2 (12) were collected at Bai Hokou and Mongambe, respectively. Fecal samples were obtained non-invasively immediately after defecation during focal follows of individual habituated gorillas. Samples from two unhabituated gorilla groups were collected only once from the gorilla nest sites to avoid duplication: U1 (11) near Bai Hokou and U2 (11) between two research camps. A number of samples and the information about individual gorillas and appropriate gorilla group are listed in Table S1. Data regarding sex and age of individual gorillas was available only for individuals from habituated groups and since we worked also with unhabituated gorillas, this information was excluded. Nest site samples were collected early in the morning, ensuring that only relatively fresh feces were obtained within 3 h from the time the gorillas had left the nests as assessed by expert trackers. The samples were collected from within the core of the feces and placed in two collection tubes, containing RNAlater® (Thermo Fisher Scientific, Waltham, MA) and 4% formaldehyde for microbiome and parasitological analyses, respectively. The samples were kept at room temperature for up to a month before transport to the Department of Pathology and Parasitology, University of Veterinary and Pharmaceutical Sciences Brno, Czech Republic (UVPS), where the samples in RNAlater® were stored at -20°C and samples in 4% formaldehyde were stored at room temperature until coprological examination. Extractions of genomic DNA were performed at the Carl R. Woese Institute for Genomic Biology, University of Illinois at Urbana-Champaign, USA. Resulting genomic DNA was shipped to the J. Craig Venter Institute, Rockville, MD, USA where DNA sequencing analyses were performed. The research adhered to the legal requirements of the Central African Republic and research protocol of DSPA. Importation of the samples to the EU was approved by the State Veterinary Authority of the Czech Republic.

### Parasitological analyses

Each sample was homogenized and strained through a sieve into Falcon conical tubes (50 ml). Samples were diluted with water up to 50 ml volume and centrifuged for 5 min at 2,000 rpm. The sediment was weighed and re-suspended to 10 ml with 4% formaldehyde. For coprological examinations, Sheather's flotation with modified sugar solution was used (specific gravity 1.33) (Sheather, 1923).

A modified simple sedimentation method (Kassai, 1999; Zajac and Conboy, 2012) was used for thorough examination and quantification of strongylid eggs. Protozoans are difficult to quantify and most of those occurring in studied gorillas are not pathogenic or even mutualistic. Strongylid nematodes with pathogenic potential are usually present in most of, if not all, examined fecal samples, so further quantification was needed in order to evaluate their impact on the GIM. Each fecal suspension (2 ml) was placed into an Eppendorf tube and centrifuged for 2 min at 1,500 rpm. The supernatant was discarded, and the remaining sediment transferred onto a microscopic slide using a micropipette, covered with a cover-glass and examined using a light microscope. The strongylid eggs present on the slide were counted. This procedure was repeated until all the sediment (from 2 ml of the solution) was examined. The number of eggs per gram of sediment (EPG) was calculated using following equation: EPG = N/(m/5) where N = number of eggs and m = weight of the whole sediment.

We reported parasite presence/absence and for strongylid nematodes, also the intensity of infection expressed as EPG. Although the number of parasite eggs shed in feces may not be linearly correlated with the intensity of infection (i.e., the number of individuals of a particular parasite species in a single infected host Bush et al., 1997) due to high variability in individual and temporal egg output (Anderson and Schad, 1985; Warnick, 1992), several studies did find a linear relationship between egg counts and the number of adult worms in the hosts (Cabaret et al., 1998; Seivwright et al., 2004), and egg output is routinely used to characterize parasite infections in domestic animals (Piekarska et al., 2013; Nielsen et al., 2014), humans (Hodges et al., 2011; Mekonnen et al., 2013), and also in wild animals, including non-human primates (Müller-Graf et al., 1996; Chapman et al., 2006; González-Hernández et al., 2014; Burgunder et al., 2017). Thus egg counts can provide a quantitative description of infection and be used as a proxy for intensity of parasite infection in non-invasive studies of endangered non-human primate populations from which individuals cannot be removed, but the results should be interpreted cautiously owing to variable egg excretion. Negative samples may possibly include samples with very low parasite intensities as given by limitations of coprological methods. With exception of *Mammomonogamus* found in our samples, the exact determination of strongylids to the genus or species level on the basis of egg morphology is unreliable. However, previous morphological and molecular analyses of L3 larvae developed in coprocultures (Kalousová and Hasegawa, unpublished data), Hasegawa et al. (2014) showed that the thin-walled strongylid eggs belonged to either the genus *Oesophagostomum* or *Necator*. Therefore we divided the strongylid eggs found in gorilla samples into two categories: (i) *Necator/Oesophagostomum* and (ii) *Mammomonogamus*.

### Microbial community analyses

Genomic DNA from fecal samples was extracted using the Power Soil DNA Isolation Kit (MoBio Laboratories Inc., Carlsbad, CA, USA) according to the manufacturer's protocol. The V1-V3 region of the 16S rRNA gene was PCR amplified (35 cycles: at 95°C for 30 s, at 55°C for 30 s and at 72°C for 30 s) using primers 27f (5′-AGAGTTTGATYMTGGCTCAG-3′, corresponding to nucleotides 27-47 of the *Escherichia coli* 16S rRNA gene) and 534r (5′-ATTACCGCGGCTGCTGGCA-3′, corresponding to nucleotides 534-515 of the *E. coli* 16S rRNA gene). Amplicon purification was done using the QIAquick PCR Purification Kit (QIAGEN, Germany). Amplicons were multiplexed and pyrosequenced using 454 FLX-Titanium technology as described previously (Gomez et al., 2015). Briefly, before following the mothur 454-SOP (Schloss et al., 2011), reads were quality filtered with an in-house perl script according to the following criteria: minimum and maximum length of 200 and 535 nt respectively; maximum number of homopolymers of 6; keeping all reverse and forward reads; removing all primer sequences and reads below *Q* = 35 over 50 bp windows. Unique sequences were aligned against the silva reference alignment database and chimeras were detected using uchime (Edgar et al., 2011) and removed. Sequences were then classified taxonomically using a Bayesian classifier approach implemented by mothur and reference files from the Ribosomal Database Project (RDP) (Wang et al., 2007) with a minimum cut-off of 80%. Then sequence reads with hits corresponding to unknown, mitochondria, chloroplasts, eukaryotes and archaea were eliminated. The remaining reads were clustered denovo using ModalClust. Reads sharing ≥ 97% 16S rRNA sequence complete-linkage similarity with the most abundant sequence were binned into an operational taxonomic unit (OTU). Taxonomic profiles were determined using the RDP Classifier (Wang et al., 2007) and the phylotype function within mothur. The QC script described above is designed in a way all spurious sequences are discarded and hence the possibility of overestimating diversity is reduced. Besides the astringent processing of the mothur 454 SOP (which emphasizes removal of spurious sequence reads), we removed OTUs present in fewer than 3 individuals and detected fewer than 5 times across the entire dataset to avoid possible 454 sequencing artifacts.

### Statistical analyses

All statistical analyses were performed using the R statistical computing language v. 3.1.2 (R Development Core Team, 2008). UniFrac distance matrices, which take into account patterns of GIM variation based on phylogenetic relationships between community taxa, were calculated in QIIME (Caporaso et al., 2010). To evaluate differences in fecal microbiome of individual samples (individuals) positive and negative for particular parasite, relative abundance of each OTU based on Bray-Curtis dissimilarity matrices were used to perform non-metric multidimensional scaling (NMDS), permutational analysis of variance (PERMANOVA), and analysis of similarities (ANOSIM) in the *vegan* package (Oksanen et al., 2013). *Vegan* package was also used to calculate Shannon diversity indices, rarefied richness analysis, and analysis of fecal microbiome dispersion in multivariate space to compare the fecal microbiome diversity between individual samples positive and negative for particular parasite. Shannon diversity indices were used to evaluate the relationships between fecal microbiome diversity and egg counts of *Necator/Oesophagostomum* and *Mammomonogamus*. A heatmap showing the differences in abundance of the most abundant bacterial genera was drawn using the *gplots* package (Warnes et al., 2016). Bacterial indicator taxa in *Entamoeba* positive and *Entamoeba* negative individuals were identified using indicator species analysis (Dufrêne and Legendre, 1997) in the *labdsv* R package (Roberts, 2013) and confirmed using Random forest classifier in the package *randomForest* (Liaw and Wiener, 2002). These analyses were performed at the microbial genus, family, and phylum levels for all collected samples. To detect differences in relative abundances of indicator bacterial taxa according to *Entamoeba* status, the Generalized linear model (GLM, negative binomial distribution, log link) with *Entamoeba* status as a predictor variable and gorilla social group as a covariate was fitted using *MASS* package (Venables and Ripley, 2002) to evaluate the differences in relative abundances of indicator taxa between *Entamoeba* positive and *Entamoeba* negative individuals. Boxplots, ANOVAs, and linear regression models with false discovery rate corrections were calculated using the *stats* package (R Development Core Team, 2008). Spearman's rank correlations with false discovery rate corrections were used to describe relationships between strongylid infection (egg counts) and levels of bacterial taxa using *psych* package (Revelle, 2017). The relationships between GIPs and fecal microbiome were determined based on parasite taxa presence/absence data and relative abundances respectively. All analyses of variance, including PERMANOVA, were performed on all individuals (=samples) pooled together with gorilla social group as a control variable and using presence/absence data for each detected parasite as independent variables.

### Data availability

Amplicon sequences have been deposited in the MG-RAST database under project ID 6321. The datasets generated or analyzed during this study are included in Supplementary Information files or are available from the corresponding author on reasonable request.

## Results

### Parasites detected

We detected: trophozoites of *Troglodytella/Gorillophilus, Prototapirella gorillae*, and other unidentified entodiniomorphid ciliates; cysts of *Blastocystis, Entamoeba* (including cysts with eight and four nuclei); eggs of *Strongyloides, Necator/Oesophagostomum, Mammomonogamus, Bertiella*, and Spirurida fam. gen (Table 1, Table S2). The median of the EPG for *Necator/Oesophagostomum* and *Mammomonogamus* was 22.96 EPG (Min: 0; Max: 22.96) and 3.13 EPG (Min: 0; Max: 93.75), respectively (see Table S3).

**Table 1:** Prevalence (%) of parasites detected in fecal samples of four gorilla groups.

	**H1**	**H2**	**U1**	**U2**	**All**
Unidentif. entod. ciliates	55.6	75	36.4	27.3	48.8
*Troglodytella/Gorillophilus*	100	91.7	100	100	97.7
*Prototapirella gorillae*	100	91.7	90.9	100	95.3
*Blastocystis*	0	25	0	9.1	9.3
*Entamoeba*	88.9	58.3	27.3	45.5	53.5
*Strongyloides*	11.1	8.3	0	18.2	9.3
*Necator/Oesophagostomum*	100	100	100	90.9	97.7
*Mammomonogamus*	55.6	75	63.6	63.6	65.1
*Bertiella*	0	8.3	0	0	2.3
Spirurida fam. gen.	33.3	66.7	36.4	18.2	39.5

### Parasitic infections and gastrointestinal microbiome

**Figure 1:**
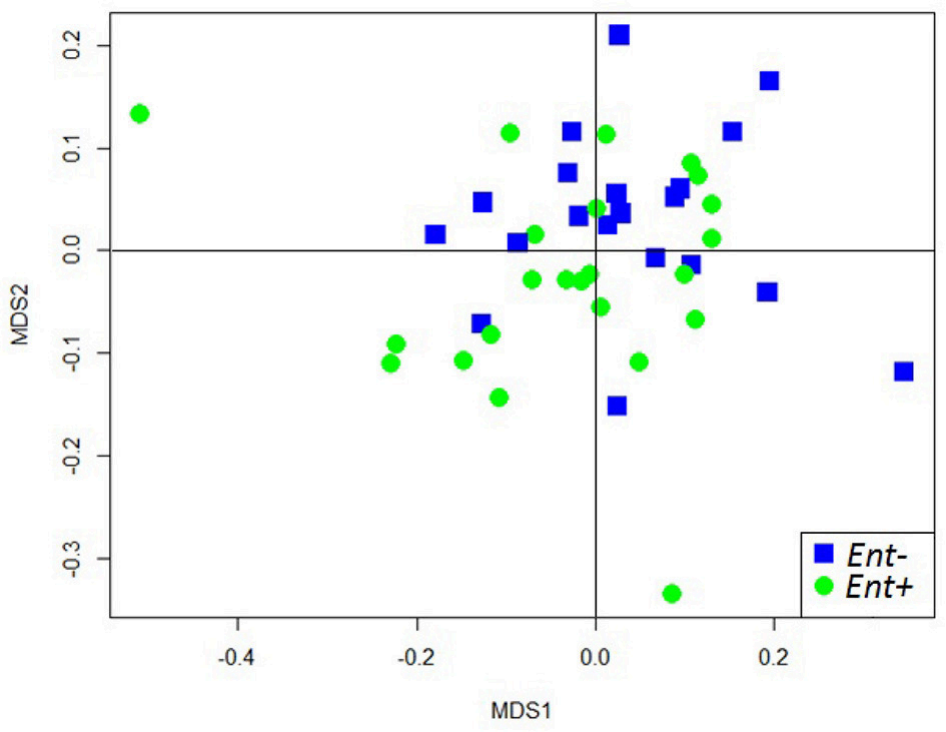
NMDS plot of unweighted UniFrac distances of fecal microbiome composition of individual gorillas colored by *Entamoeba* status.

We obtained a median sampling depth of 6,458 reads per gorilla fecal sample (Min: 2,114; Max: 116,361) after sequence quality control (for further detail see Gomez et al., 2015, 2016b). The median of the raw reads was 6,559 (Min: 2,204; Max: 117,457) per sample. The total number of reads obtained in total was 421,553 and 416,624 after quality filtering. We found weak differences in the overall GIM composition only between *Entamoeba* positive (*Ent+*) and *Entamoeba* negative (*Ent-*) individuals (PERMANOVA for OTU relative abundances, based on Bray-Curtis dissimilarity matrices: Pseudo-F = 1.985, *R*^2^ = 0.043, *p* = 0.009, Table 2, Figure S2). Both weighted and unweighted UniFrac distance matrices also indicated differences in GIM composition between *Ent+* and *Ent-* individuals (PERMANOVA: Pseudo-F = 1.668, *R*^2^ = 0.037, *p* = 0.053; Pseudo-F = 1.371, *R*^2^ = 0.031, *p* = 0.006, for weighted and unweighted UniFrac distances respectively; Table 2, Figure 1). We found no differences in overall GIM composition between *Ent+* and *Ent-* gorillas according to ANOSIM analyses: Bray-Curtis dissimilarity matrices (*R* = 0.091, *p* = 0.017), weighted UniFrac (*R* = 0.040, *p* = 0.107), and unweighted UniFrac (*R* = 0.010, *p* = 0.296).

**Table 2:** Differences in fecal microbiome at the OTU level between individuals positive and negative for particular parasite based on PERMANOVA with gorilla social group as controlled variable.

**Covariate**	**Bray-Curtis dissimilarity matrices**			**Weighted UniFrac**			**Unweighted UniFrac**		
	**Pseudo-F**	**R2**	**p**	**Pseudo-F**	**R2**	**p**	**Pseudo-F**	**R2**	**p**
*Entamoeba*	1.985	0.043	0.009	1.668	0.037	0.053	1.371	0.031	0.006
Group	2.101	0.136	0.002	1.776	0.118	0.002	1.515	0.103	0.001
Unidentif. entod. cilliates	1.240	0.027	0.197	1.183	0.026	0.248	1.119	0.026	0.108
Group	2.180	0.143	0.001	1.861	0.125	0.003	1.597	0.110	0.001
*Blastocystis*	1.052	0.023	0.366	0.565	0.013	0.913	0.865	0.021	0.920
Group	2.193	0.144	0.001	1.806	0.123	0.001	1.545	0.107	0.001
*Strongyloides*	1.026	0.023	0.419	1.001	0.225	0.416	1.117	0.025	0.143
Group	2.176	0.143	0.001	1.810	0.122	0.003	1.572	0.108	0.001
*Mammomonogamus*	0.628	0.014	0.947	0.676	0.015	0.840	0.943	0.022	0.706
Group	2.187	0.145	0.001	1.841	0.125	0.004	1.597	0.110	0.001
Spirurida fam. gen.	0.811	0.018	0.747	0.581	0.013	0.941	0.962	0.022	0.604
Group	2.153	0.143	0.002	1.900	0.129	0.003	1.602	0.111	0.001

**Figure 2:**
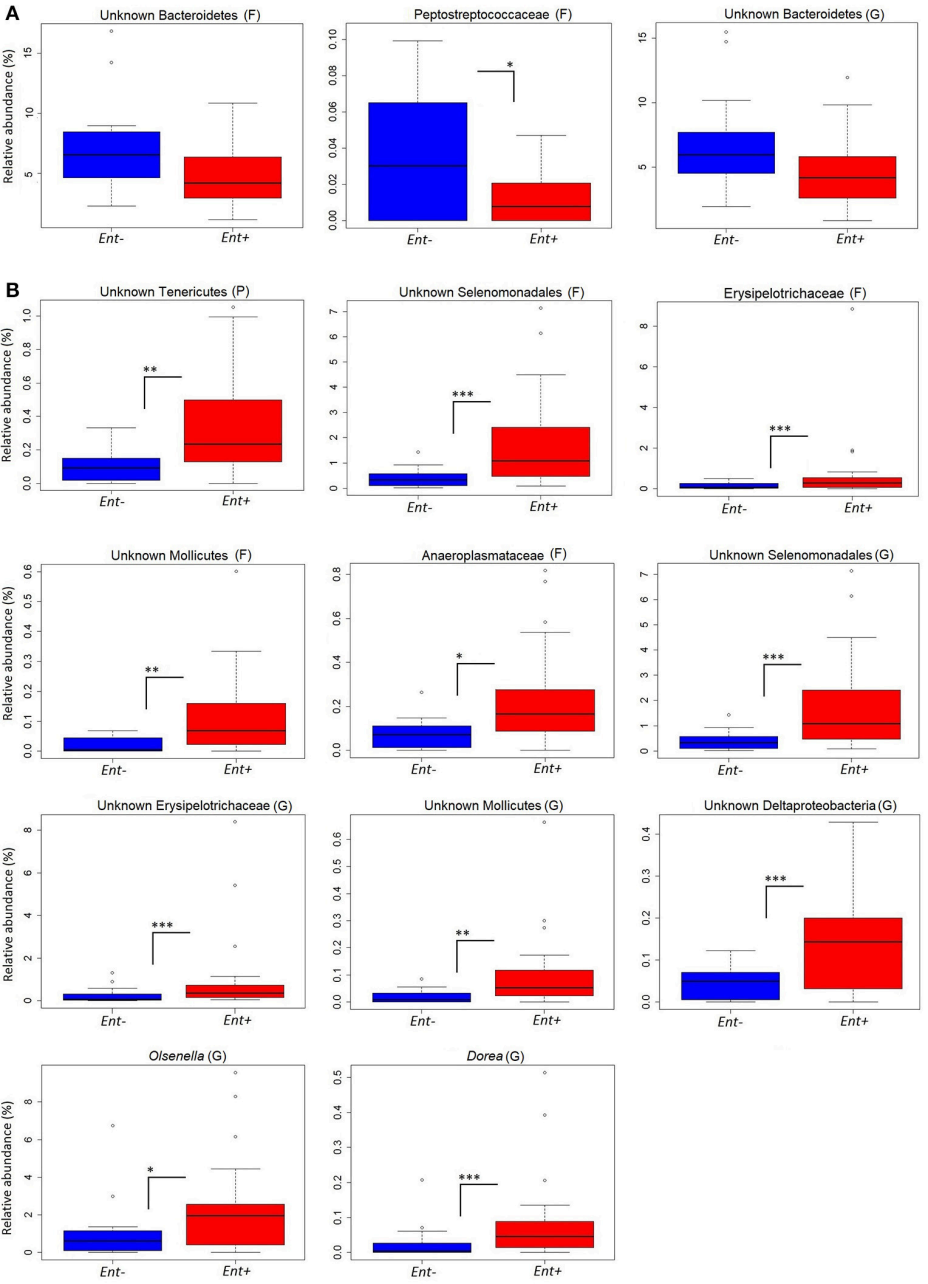
Relative abundances of indicator taxa based on *Entamoeba* status. Significant differences in relative abundances (%) of indicator taxa in *Ent-* **(A)** and *Ent+* **(B)** individuals based on GLM with negative binomial distribution: ***0.001, **0.01, *0.5. Taxonomic rank: P, phyla; F, family; G, genus.

We detected significant differences in relative abundances of indicator taxa, detected by Species indicator analysis and Random forest classifier, in *Ent-* and *Ent+* individuals based on GLM (negative binomial distribution, log link) with *Entamoeba* status as a predictor variable and gorilla social group as a covariate (Table 3, Figure 2). Specifically, at the phylum level, unknown Tenericutes (GLM: *F* = 2.992, *p* = 0.003) reached higher levels in *Ent*+ individuals. At the family level, Peptostreptococcaceae reached significantly higher levels in *Ent*− individuals (GLM: *F* = −2.559, *p* = 0.010), while unknown Selenomonadales (GLM: *F* = 3.935, *p* < 0.001), Erysipelotrichaceae (GLM: *F* = 3.505, *p* < 0.001), unknown Mollicutes (GLM: *F* = 3.282, *p* = 0.001), and Anaeroplasmataceae (GLM: *F* = 2.473, *p* = 0.013) reached higher levels in *Ent+* individuals. At the genus level, unknown Selenomonadales (GLM: *F* = 3.697, *p* < 0.001), unknown Erysipelotrichaceae (GLM: *F* = 2.815, *p* = 0.005), unknown Molicutes (GLM: *F* = 3.260, *p* = 0.001), unknown Deltaproteobacteria (GLM: *F* = 4.217, *p* < 0.001), *Olsenella* (GLM: *F* = 2.852, *p* = 0.004), and *Dorea* (GLM: *F* = 3.167, *p* = 0.002) reached significantly higher levels in *Ent+* individuals. Three out of six indicator taxa on genus level belonged among the 26 most abundant bacterial genera (Figure S3). Although *Entamoeba* status significantly predicted the abundance of these taxa, social group membership was also a significant factor in some of these taxa (GLM: *p* < 0.05; data not shown). We did not find significant differences in relative abundances of any other bacterial taxa between individuals positive or negative for other detected parasites (data not shown).

**Table 3:** Indicator taxa with mean relative abundances in *Ent-* and *Ent+* individuals.

**Taxonomic rank**	**Indicator taxa in *Ent*−**	**Indicator value**	**Probability**	**Mean relative abundance % (SD)**	
				***Ent*−**	***Ent*+**
Family	Unknown (Bacteroidetes)	0.599	0.022	6.547 (3.632)	4.192 (2.556)
Family	Peptostreptococcaceae	0.535	0.018	0.030^*^ (0.032)	0.007 (0.014)
Genus	Unknown (Bacteroidetes)	0.595	0.04	5.962 (3.544)	4.198 (2.834)
**Taxonomic rank**	**Indicator taxa in** ***Ent+***	**Indicator value**	**Probability**	**Mean relative abundance % (SD)**	
				***Ent*−**	***Ent*+**
Phyla	Unknown (Tenericutes)	0.745	0.002	0.091 (0.086)	0.233^**^ (0.321)
Family	Unknown (Selenomonadales)	0.837	0.001	0.275 (0.371)	1.056^***^ (2.030)
Family	Erysipelotrichaceae	0.809	0.026	0.064 (0.172)	0.284^***^ (1.826)
Family	Unknown (Mollicutes)	0.73	0.001	0.004 (0.026)	0.068^**^ (0.139)
Family	Anaeroplasmataceae	0.723	0.003	0.071 (0.066)	0.166^*^ (0.232)
Genus	Unknown (Selenomonadales)	0.825	0.002	0.325 (0.362)	1.083^***^ (1.961)
Genus	Unknown (Erysipelotrichaceae)	0.818	0.007	0.077 (0.345)	0.359^**^ (1.977)
Genus	Unknown (Mollicutes)	0.734	0.002	0.009 (0.024)	0.052^**^ (0.147)
Genus	Unknown (Deltaproteobacteria)	0.675	0.005	0.049 (0.038)	0.144^***^ (0.105)
Genus	*Olsenella*	0.663	0.047	0.625 (1.518)	1.952^**^ (2.577)
Genus	*Dorea*	0.646	0.006	0.005 (0.048)	0.046^**^ (0.127)

*Significant differences based on GLM with negative binomial distribution*,

*** *0.01*,

** 0.05, and

* *0.1*.

**Figure 3:**
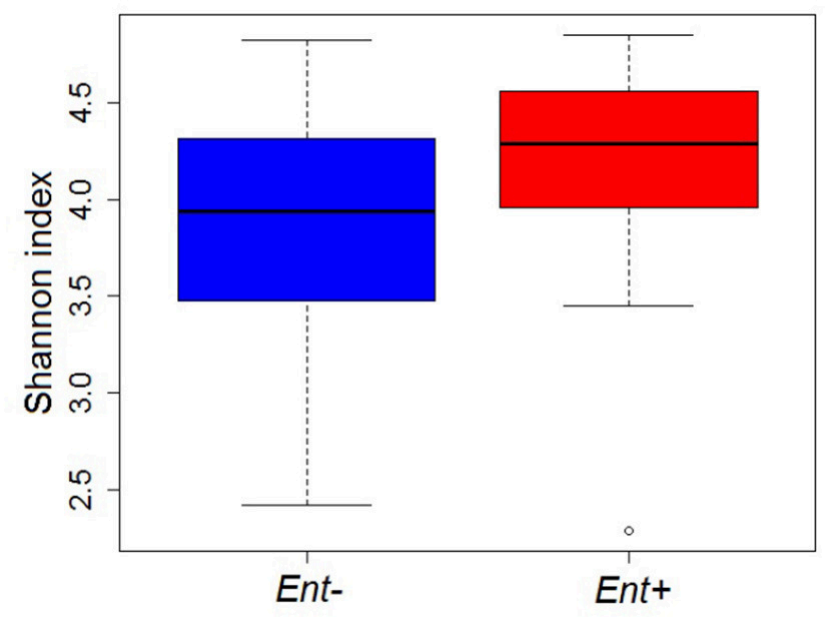
GIM diversity (Shannon diversity indices) in *Ent+* and *Ent-* individuals. ANOVA: *p* = 0.101.

GIM diversity (Shannon diversity index) did not significantly differ between *Ent+* and *Ent-* individuals, even though they reached higher values in *Ent+* individuals (ANOVA: *F* = 2.823, *p* = 0.101; Figure 3). Moreover, we found no significant differences between *Ent*+ and *Ent*- individuals based on rarefied richness analysis (ANOVA: *F* = 1.129, *p* = 0.295). Analyses of multivariate dispersion of communities or inter-individual variation within groups also did not show differences between *Ent*+ and *Ent*- individuals (ANOVA: *F* = 0.047, *p* = 0.829; Figure S4).

**Figure 4:**
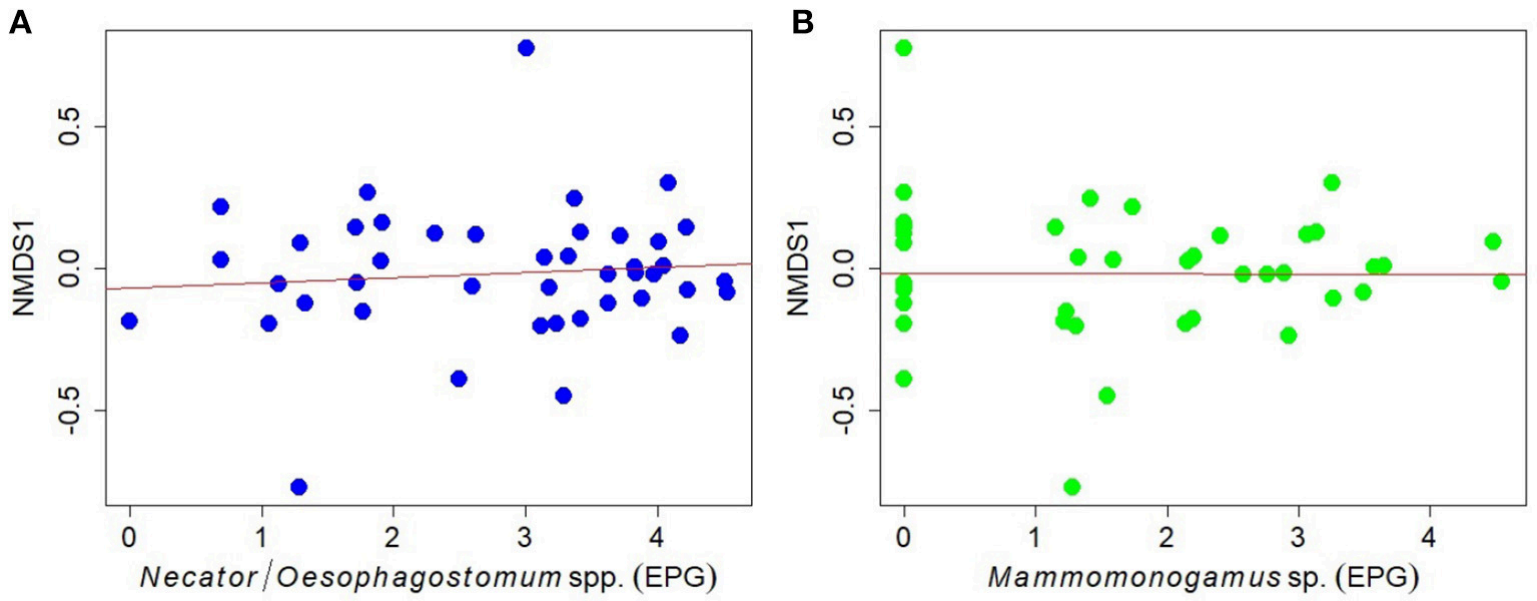
Relationships between the egg counts of **(A)** *Necator/Oesophagostomum* spp., **(B)** *Mammomonogamus* sp. (natural log-transformed data) and overall GIM community (as described with the first NMDS axis, NMDS1) in individual gorillas.

Using linear regression models we found no relationships between egg counts of *Necator/Oesophagostomum* and *Mammomonogamus* (EPG) and overall fecal microbiome composition, as described by the first NMDS axis, NMDS1 (*R*^2^ = 0.009, *t* = 0.618, *p* = 0.540; *R*^2^ < 0.001, *t* = 0.025, *p* = 0.974, for *Necator/Oesophagostomum* and *Mammomonogamus* respectively; Figures 4A,B). The results were also confirmed by Spearman's rank correlations (*Necator/Oesophagostomum*, ρ = −2.795, *p* = 0.069; *Mammomonogamus*, ρ = −0.180, *p* = 0.247). We found weak relationships only between relative abundances of members of phylum Verrucomicrobia, family Verrucomicrobia subdivision 5 and *Necator/Oesophagostomum* egg counts based on Spearman's rank correlations and linear regression models (Figures S5A,B), but when we applied false discovery rate corrections, we did not find the significant results anymore, even if we defined the fdr-q value threshold as 0.1 (data not shown). The relationships between relative abundances of other detected bacterial taxa and *Necator/Oesophagostomum* egg counts were not statistically significant (data not shown). We did not find any significant relationship between relative abundances of any bacterial taxa and *Mammomonogamus* egg counts using Spearmans rank correlation analyses and linear regression model (data not shown).

We found no relationship between fecal microbiome diversity (Shannon diversity indices) and egg count of *Necator/Oesophagostomum* (Spearman's rank correlation: ρ = −0.218, *p* = 0.161; linear regression model: *R*^2^ = 0.005, *t* = −1.434, *p* = 0.159, Figure S6) or *Mammomonogamus* (Spearman's rank correlation: ρ = −0.010, *p* = 0.949; linear regression model: *R*^2^ < 0.001, *t* = 0.095, *p* = 0.093, Figure S6).

## Discussion

Given the evolutionary relationships between non-human primates and humans, studying associations between parasites and gut bacteria in great apes may help to understand current configurations of the human gut microbiome in the context of health and disease. Although the effect of GIP infection on the bacterial community composition of western lowland gorillas appears to be weak, our data show evidence that presence of *Entamoeba* spp. is associated with the abundance of specific bacterial taxa in the gorilla gastrointestinal tract. The mechanism behind these observations is unknown, however, this may be a reflection of either direct or indirect interaction between entamoebas, the GIM, and the host immune system. The genus *Entamoeba* contains both pathogenic and commensal species (Verweij et al., 2003; Stensvold et al., 2010). Although much is known about parasitic *E. histolytica*, the causative agent of amoebic dysentery in humans, and its impact on GIM (Rani et al., 2006; Galván-Moroyoqui et al., 2008; Verma et al., 2012; Burgess and Petri, 2016), there is no information about the non-pathogenic *Entamoeba* species and their relationship with host's GIM. In severe cases, *E. histolytica* trophozoites colonize the large intestine, especially the cecal and sigmoidorectal regions, where they feed on cellular debris and certain species of bacteria, allowing others to proliferate or inducing a host immune response that differentially affects the survival of different microbes (Morton et al., 2015; Burgess and Petri, 2016). It has been shown that direct competition by intestinal commensals can provide protection from invading pathogens, thus a disturbance of the normal GIM may result in increased susceptibility to pathogens (Hayes et al., 2010; Buffie and Pamer, 2013). Moreover, it is assumed that a specific gut microbiota predisposes an individual to *Enatmoeba* colonization (Bracha et al., 1982; Galván-Moroyoqui et al., 2008).

Our data indicate that the presence of *Entamoeba* is not associated with heterogeneity and dispersion of the fecal microbiome in lowland gorillas. Thus, either larger sample sizes may be needed to detect such differences or the effects of *Entamoeba* infections in the fecal microbiome differ between humans and non-human primates since a higher alpha diversity has been previously detected upon infection with *Entamoeba* spp. in hunter-gatherer and agricultural human populations (Morton et al., 2015). Nevertheless, we observed that in most cases, with the exception of family Peptostreptococcaceae, *Ent+* individuals have a higher abundance of taxa that may play an important role in nutrition, and metabolism for the host (e.g., fiber degraders, carbohydrate and glucose fermenters) and taxa that seem to be a signature of the fecal microbiome of gorillas even if they belong among minor taxa (e.g., *Olsenella, Dorea*, and Verrucomicrobiacea) (Gomez et al., 2015, 2016b). We detected significant changes in members of dominant mammalian GIM phyla: Firmicutes, and Actinobacteria (Lay et al., 2005) associated with *Entamoeba* infection. We also found differences in members of Bacteroidetes, specifically unknown Bacteroidetes, nevertheless those were not significant. However, lower levels of members of the genus *Bacteroides*, which are important contributors to carbohydrate metabolism, nutrition and health of human and animals, have been previously observed in *Ent+* people (Rani et al., 2006; Verma et al., 2012; Morton et al., 2015). Several genera belonging to Firmicutes reached significantly higher levels in *Ent+* gorillas. For instance, *Dorea*, a common member of the human GIM, and important short-chain fatty acid producing bacteria (Guinane and Cotter, 2013; Schnorr et al., 2014), was more abundant in *Ent+* individuals. A higher abundance of the Anaeroplasmataceae family was found in *Ent+* individuals. These bacteria are known contributors to fermentative mechanisms in environmental micro-ecosystems (Koike et al., 2003); thus, its abundance may be beneficial for the host in the context of foraging efficiency. Members of family Erysipelotrichaceae, previously associated with several diseases in humans (Kaakoush, 2015), also reached higher levels in *Ent+* individuals, however studies examining the direct impact that changes in the abundance of Erysipelotrichaceae have on the host are still required. *Olsenella*, a common member of GIM of great apes (Gomez et al., 2015; Moeller et al., 2016) reached higher relative abundances in *Ent+* individuals. This genus was found to be more abundant in humans suffering from soil-transmitted helminths (Rosa et al., 2018). *Olsenella* has been previously associated with a reduction in gut inflammation (Wang et al., 2015), suggesting that its association with *Entamoeba* infection may potentially have positive side effects on the gorilla gut health.

Our observations raise questions about the natural occurrence of *Entamoeba* infections in the gorilla gastrointestinal ecosystem or the potential advantage of harboring these amoebas in terms of nutrition. Future studies should explore specific molecular interactions between hosts and their resident parasites and bacteria, seeking to determine if parasites help to retain nutritionally useful bacteria and displace those that are not essential or are harmful. Moreover, the prevalence and apparently harmless nature of some parasites (e.g., most of *Entamoeba* spp.) in gorillas must be tested molecularly, by specifically targeting the presence and expression of known parasite-associated virulence factors. Since large diversity of *Entamoeba* spp. has been observed in wild great apes (Vlčková et al., 2018), the molecular identification of particular *Entamoeba* species/genotypes may help to discriminate among particular parasite-bacteria interactions involving selected species, and the way they impact the GIM. Moreover, since many of the detected bacterial taxa, which significantly differed in studied individuals based on *Entamoeba* status belong to unknown species, it is difficult to evaluate the effect of these differences on the host. Due to the high complexity of the system, the metabolomic analyses should be included in the further studies to evaluate possible changes in the overall functionality of the GIM.

We did not find any associations between bacterial relative abundances as well as fecal microbiome diversity and intensity of *Necator/Oesophagostomum* and *Mammomonogamus* infection. However, our results could be affected by the inability to distinguish eggs of strongylid nematodes using the coprological methods as the life cycles and pathogeneses of these strongylids are different. Recently, a hidden diversity in the strongylid nematode communities in this gorilla population has been revealed by implementation ITS-2 metabarcoding at the Illumina MiSeq platform (Pafčo et al., 2018). Such approach allowed the detection of rare, otherwise overlooked taxa such as *Ternidens diminutus* and *Libyostrongylus* sp. present in a small number of samples and represented by a low numbers of reads. In future research, advanced molecular tools need to be implemented also to study GIP diversity to assess the associations between strongylids and GIM. Ensuing inflammatory responses from the host to strongylid infections may induce a gut environment with microbe-selective properties, which is further modulated by diet. As we did not record any clinical signs of strongylid infections in the studied gorillas, we may assume that the levels of *Necator/Oesophagostomum* infection may be too low and their abundance in the gorilla GIM may be facilitated by immune adaptations to pathogen exposure. It is also possible that strongylids may induce modifications of the GIM at the site of infection (e.g., duodenal mucosa for hookworms) that are not reflected in the fecal microbiome composition (Cooper et al., 2013). Along these lines, and in light of the potential therapeutic application of hookworms and suggestions of using chronic hookworm infection as immunomodulatory therapy to counteract detrimental immune responses (Daveson et al., 2011), the contention of parasites as a ubiquitous fraction of the GIM, interacting in synergy with specific GIM signatures in non-human primates and traditional populations should be explored.

Here, we examined the associations between GIP infections and the GIM composition of wild lowland gorillas. Although we did not observe notable changes in global bacterial community profiles in connection with GIP infections, we found significant relationships between *Entamoeba* infections and abundances of certain gut bacterial taxa. These changes could be mediated by complex interactions of the GIPs with the host ecology (diet), the host immune machinery, and the other microbiota. To gain further insight into the specific nature of these interactions, it will be necessary to apply additional molecular approaches to assess GIP diversity and function at the mucosal level to understand how the host immune system reacts to the GIPs and their virulence factors, with subsequent consequences for gut bacterial communities. Likewise, the role of diet in this interaction should be clarified from a molecular perspective in terms of metabolic impact on the GIPs and the other microbiota. For more complex analyses adding other factors affecting fecal microbiome of great apes, e.g., sex and age would be also beneficial, nevertheless, it would require studying captive or larger number of habituated animals. Moreover, future work may be directed into analyzing the evolutionary correlates of GIP infections for health and disease in humans and great apes. Our results can have implications for understanding host-microbe interactions and gorilla welfare in both wild and captive conditions. Absence of GIP in captive apes may be responsible for particular health or digestive problems (together with other factors like diet), on the other hand, it is well-known that particular GIP in captive apes can reach higher intensities than in the wild and infected animals suffer from clinical outcomes which are rarely observed in the wild.

## Author contributions

KV and AG conceived the initial project design, with inputs from KP; BP KP and AT designed the field study and collected the samples; KV, AG, MT performed the microbial community analyses; BP performed parasitological analyses; KV analyzed the data with significant contributions from AG; KP, DM, KN, BreW, BryW, CY, RS, SL provided the financial support; KV wrote the initial manuscript with significant contributions from AG and KP. All authors reviewed the manuscript.

### Conflict of interest statement

The authors declare that the research was conducted in the absence of any commercial or financial relationships that could be construed as a potential conflict of interest. The reviewer AK and handling Editor declared their shared affiliation.
