# Supplementary material for: Relationships Between Gastrointestinal Parasite Infections and the Fecal Microbiome in Free-Ranging Western Lowland Gorillas
Source: Front Microbiol. 2018 Jun 15;9:1202. doi: 10.3389/fmicb.2018.01202 (PMC6013710; doi:10.3389/fmicb.2018.01202)
Supplement: Supplementary file 1 [file Data_Sheet_1.pdf]

Supplementary materials

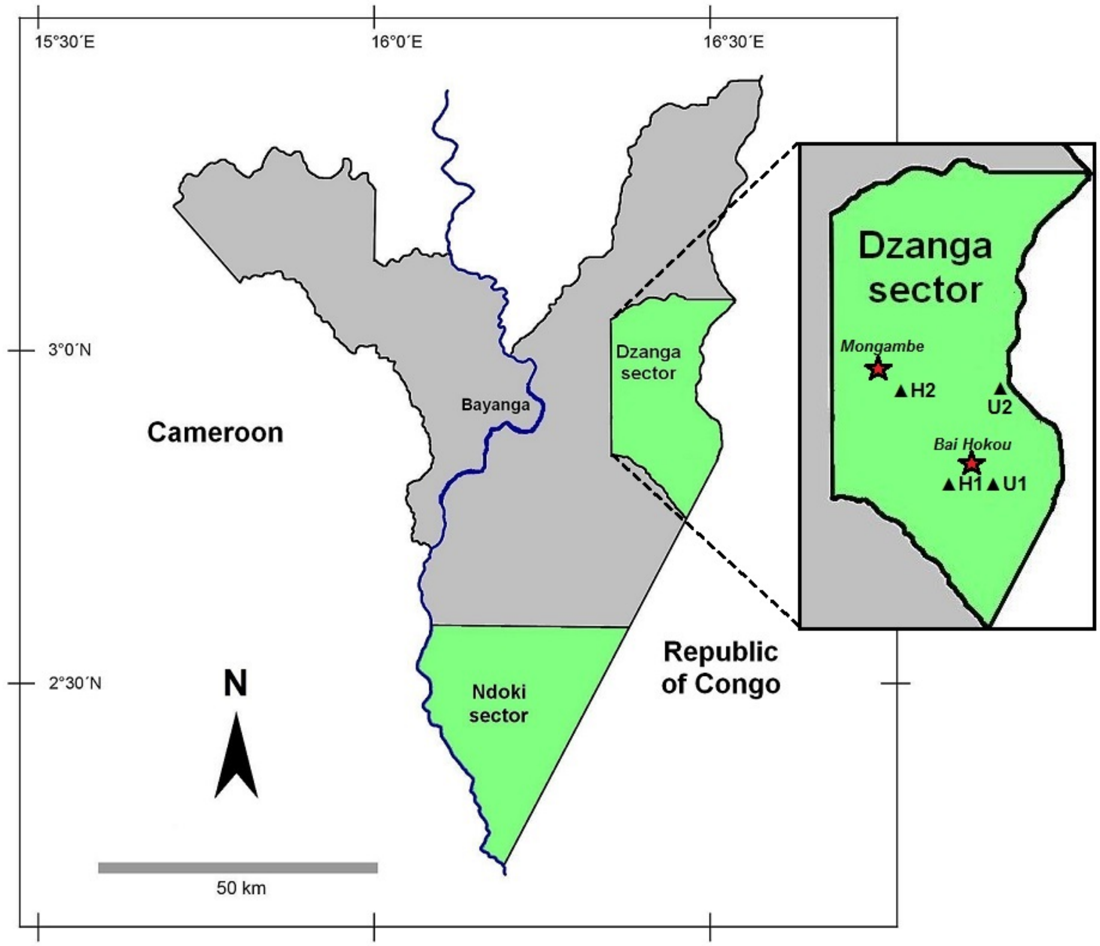

Figure S1. Map of the study site in Dzanga-Sangha Protected Areas, Central African Republic. Picture modified from Vlčková et al. (2016).

Table S1. List of fecal samples of individual gorillas from four gorilla groups

Gorilla group	Individual	Age group	Sex
U1	#1	NA	NA
U1	#2	NA	NA
U1	#3	NA	NA
U1	#4	NA	NA
U1	#5	NA	NA
U1	#6	NA	NA
U1	#7	NA	NA
U1	#8	NA	NA
U1	#9	NA	NA
U1	#10	NA	NA
U1	#11	NA	NA
H1	Bokata	Juvenile	Female
H1	Kunga	Subadult	Male
H1	Makumba	Adult	Male
H1	Malui	Adult	Female
H1	Mobangi	Juvenile	Male
H1	Mopambi	Adult	Female
H1	Mossoko	Juvenile	Female
H1	Sopo	Baby	Female
H1	Tembo	Baby	Male
H2	Elili	Adult	Female
H2	Liamba	Juvenile	Female
H2	Lungu/Kaya	Baby	NA
H2	Mambeka	Subadult	Female
H2	Mapoki	Adult	Female
H2	Mayele	Adult	Male
H2	Mopangu	Subadult	Male
H2	Mwangale	Baby	NA
H2	Ngobo	Juvenile	Male
H2	Penge	Adult	Female
H2	Sosa	Subadult	Male
H2	Yoko	Juvenile	Male
U2	#1	NA	NA
U2	#2	NA	NA
U2	#3	NA	NA
U2	#4	NA	NA
U2	#5	NA	NA
U2	#6	NA	NA
U2	#7	NA	NA
U2	#8	NA	NA
U2	#9	NA	NA
U2	#10	NA	NA
U2	#11	NA	NA

Table S2. Presence/absence of parasites detected in individual gorillas from four groups

**Gorilla group**	**Individual**	**Unident. entod. ciliates**	***Troglodytella/ Gorillophilus***	***Prototapire-lla gorillae***	***Blasto-cystis***	***Enta-moeba***	***Strongy-loides***	***Necator/ Oesophago-stomum***	***Mammo-monogamus***	***Bertiella***	**Spirurida fam. gen.**
U1	#1	0	1	1	0	0	0	1	1	0	1
U1	#2	1	1	1	0	0	0	1	0	0	0
U1	#3	0	1	1	0	1	0	1	0	0	0
U1	#4	0	1	1	0	0	0	1	1	0	0
U1	#5	1	1	1	0	0	0	1	1	0	0
U1	#6	1	1	1	0	0	0	1	1	0	1
U1	#7	1	1	0	0	0	0	1	1	0	0
U1	#8	0	1	1	0	1	0	1	1	0	0
U1	#9	0	1	1	0	0	0	1	0	0	1
U1	#10	0	1	1	0	0	0	1	0	0	0
U1	#11	0	1	1	0	1	0	1	1	0	1
H1	Bokata	1	1	1	0	1	0	1	1	0	1
H1	Kunga	0	1	1	0	1	0	1	0	0	0
H1	Makumba	1	1	1	0	1	0	1	0	0	0
H1	Malui	0	1	1	0	0	0	1	1	0	1
H1	Mobangi	0	1	1	0	1	0	1	1	0	0
H1	Mopambi	0	1	1	0	1	0	1	1	0	0
H1	Mossoko	1	1	1	0	1	0	1	1	0	1
H1	Sopo	1	1	1	0	1	0	1	0	0	0
H1	Tembo	1	1	1	0	1	1	1	0	0	0
H2	Elili	1	1	1	0	1	0	1	1	0	0
H2	Liamba	0	1	1	0	0	0	1	1	0	1
H2	Lungu/Kaya	1	0	0	0	0	1	1	0	0	0
H2	Mambeka	1	1	1	0	1	0	1	1	0	1
H2	Mapoki	1	1	1	0	1	0	1	0	0	1
H2	Mayele	1	1	1	0	1	0	1	1	0	1
H2	Mopangu	1	1	1	1	0	0	1	1	0	1
H2	Mwangale	0	1	1	0	1	0	1	1	0	1
H2	Ngobo	1	1	1	1	0	0	1	0	0	0
H2	Penge	0	1	1	0	0	0	1	1	1	1
H2	Sosa	1	1	1	0	1	0	1	1	0	1
H2	Yoko	1	1	1	1	1	0	1	1	0	0
U2	#1	0	1	1	0	1	0	1	0	0	0
U2	#2	0	1	1	0	0	0	1	1	0	0
U2	#3	1	1	1	1	0	0	1	1	0	0
U2	#4	0	1	1	0	0	0	0	1	0	0

Continued on the next page

Table S2. Presence/absence of parasites detected in individual gorillas from four groups

**Gorilla group**	**Individual**	**Unident. entod. ciliates**	***Troglodytella/ Gorillophilus***	***Prototapire-lla gorillae***	***Blasto-cystis***	***Enta-moeba***	***Strongy-loides***	***Necator/ Oesophago-stomum***	***Mammo-monogamus***	***Bertiella***	**Spirurida fam. gen.**
U2	#5	0	1	1	0	0	0	1	1	0	0
U2	#6	0	1	1	0	1	1	1	1	0	1
U2	#7	0	1	1	0	1	0	1	0	0	0
U2	#8	1	1	1	0	0	0	1	1	0	0
U2	#9	1	1	1	0	1	0	1	0	0	1
U2	#10	0	1	1	0	0	1	1	1	0	0
U2	#11	0	1	1	0	1	0	1	0	0	0

Table S3. Intensity (EPG) of infection of *Necator/ Oesophagostomum* spp. and *Mammomonogamus* sp. in individual gorillas from four groups

Gorilla group	Individual	*Necator/ Oesophagostomum*	*Mammomonogamus*
U1	#1	89.84	93.75
U1	#2	12.38	0
U1	#3	22.96	0
U1	#4	47.49	25.14
U1	#5	51.98	14.85
U1	#6	92	32
U1	#7	58.33	25
U1	#8	4.52	2.16
U1	#9	67.38	0
U1	#10	36.48	0
U1	#11	56.39	37.59
H1	Bokata	26.88	8.06
H1	Kunga	5.1	0
H1	Makumba	5.78	0
H1	Malui	63.83	17.73
H1	Mobangi	36.59	12.2
H1	Mopambi	1	4.69
H1	Mossoko	22.1	2.76
H1	Sopo	4.61	0
H1	Tembo	2.79	0
H2	Elili	12.82	20.51
H2	Liamba	29.41	8.02
H2	Lungu/Kaya	45.45	17.05
H2	Mambeka	44.78	34.83
H2	Mapoki	67.31	0
H2	Mayele	29.41	22.06
H2	Mopangu	1	3.91
H2	Mwangale	28.13	3.13
H2	Ngobo	9.09	0
H2	Penge	40.27	10.07
H2	Sosa	54.05	87.84
H2	Yoko	5.73	7.63
U2	#1	19.23	0
U2	#2	25.74	3.68
U2	#3	21.62	2.7
U2	#4	0	2.37
U2	#5	2.6	2.6
U2	#6	24.44	7.52
U2	#7	2.1	0
U2	#8	4.85	2.43
U2	#9	11.11	0
U2	#10	2.65	0
U2	#11	1.87	0

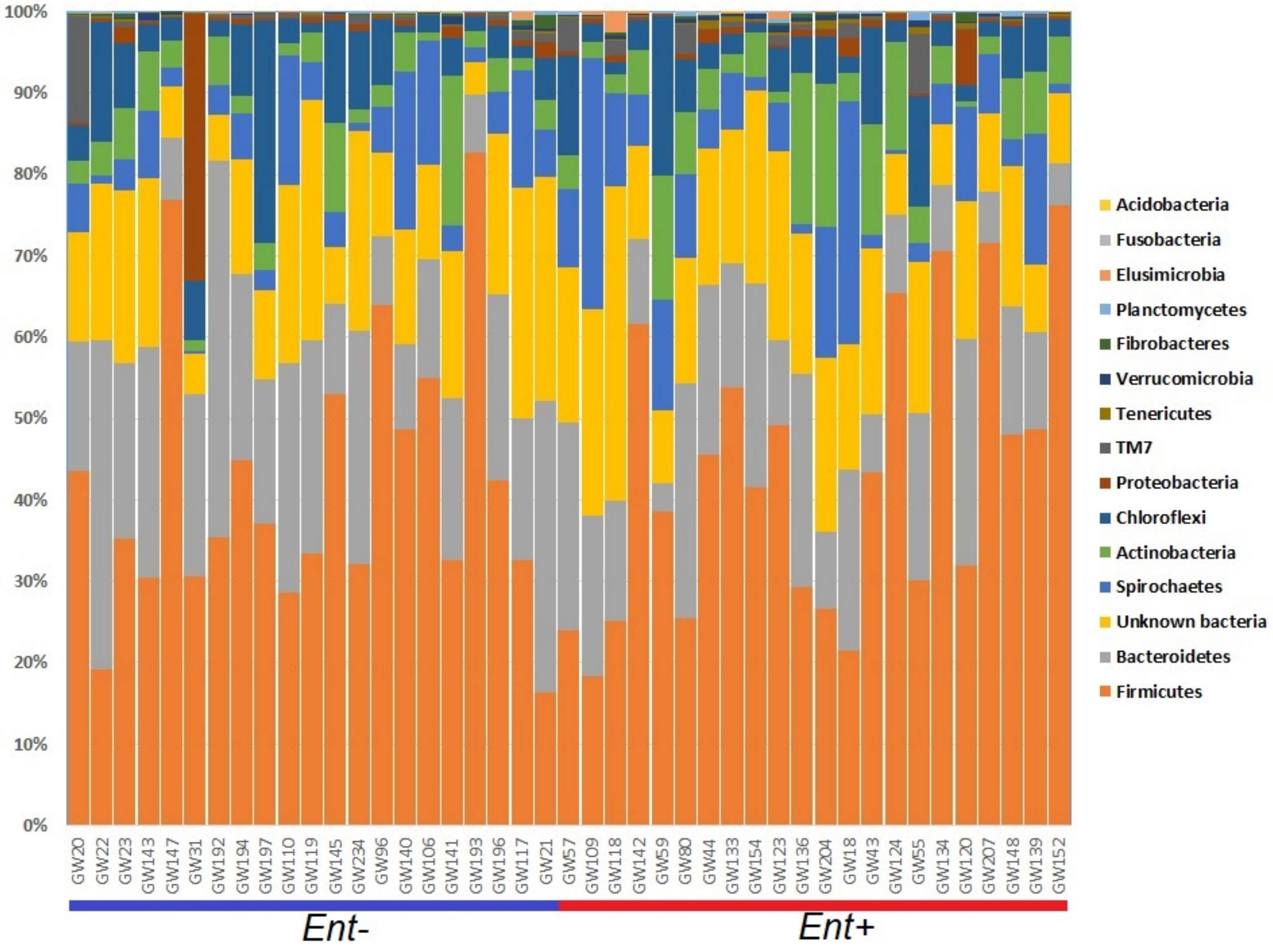

Figure S2. Relative abundances of bacterial phyla in *Ent*+ and *Ent*- gorillas

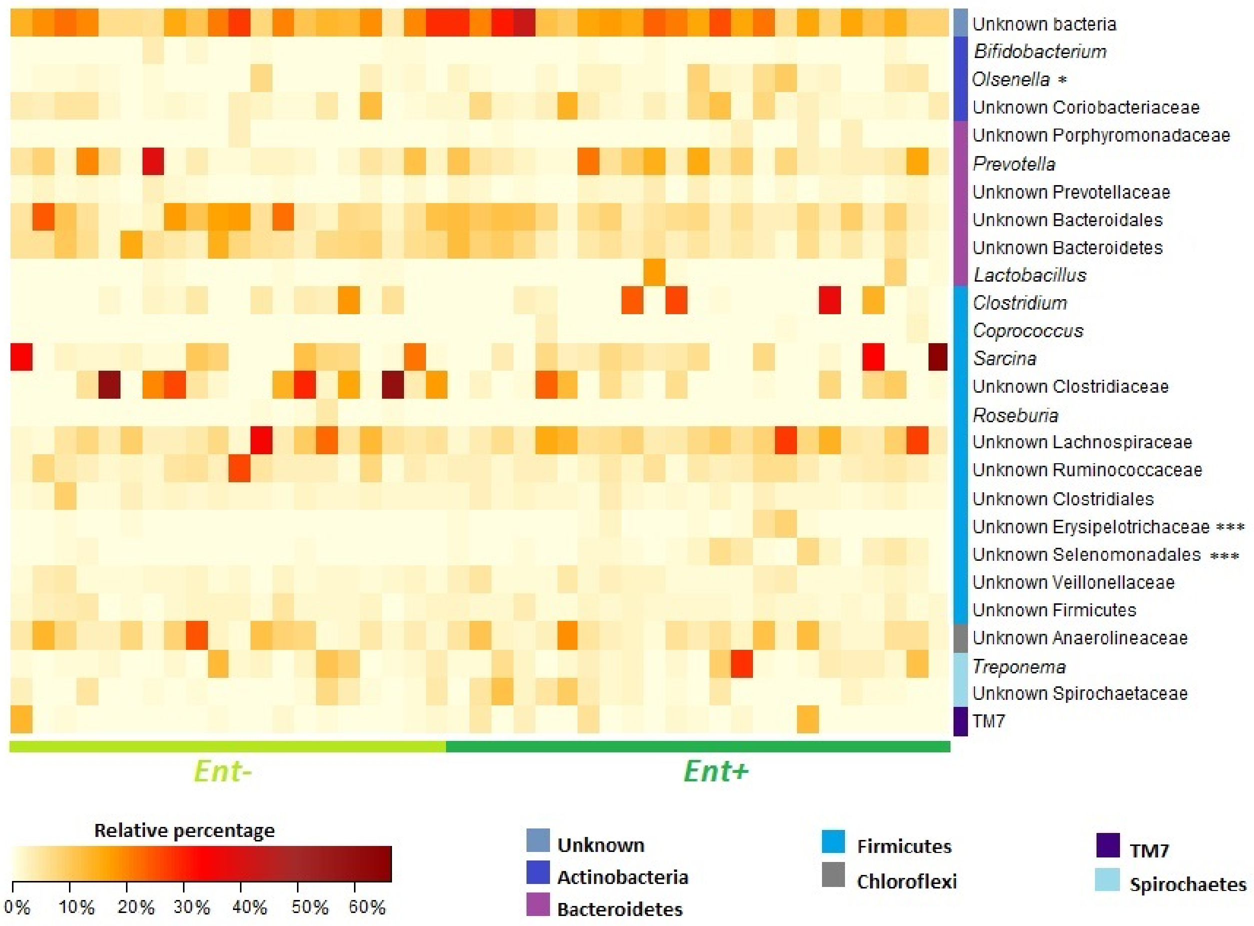

Figure S3. Heatmap showing 26 most abundant bacterial genera in *Ent-* and *Ent+* individuals. Significant differences between *Ent-* and *Ent+* individuals based on GLM with negative binomial distribution: *** 0.001, * 0.5.

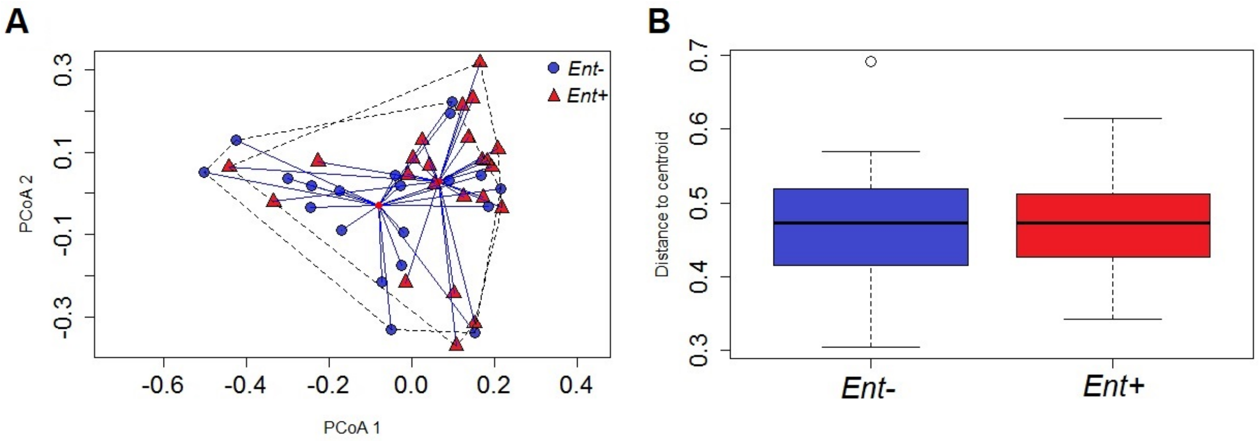

Figure S4. GIM dispersion of *Ent+* and *Ent-* individuals (**A**) in multivariate space and (**B**) in boxplots.

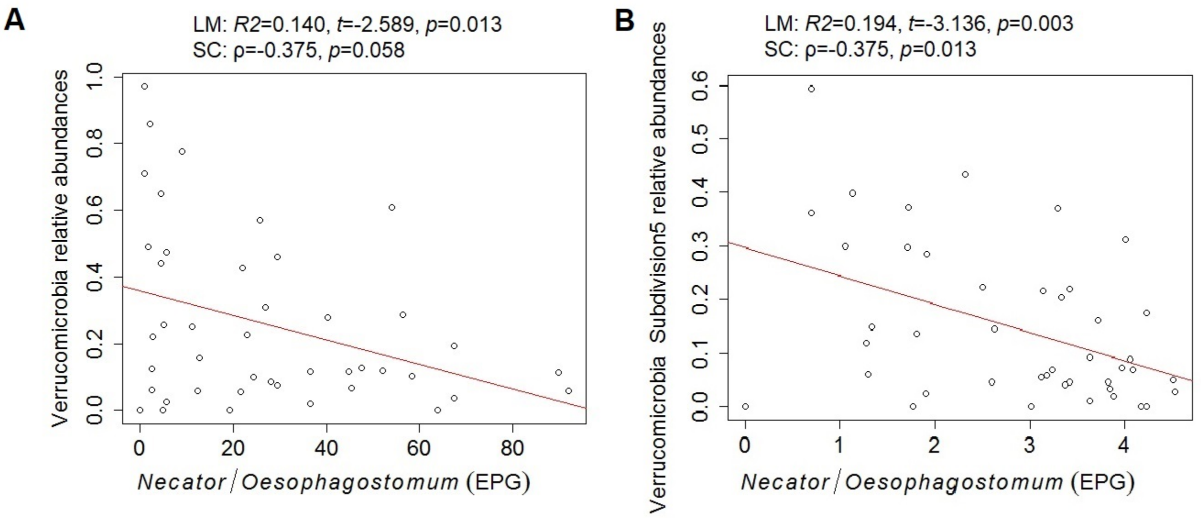

Figure S5. Linear regression model of relationships between the egg counts of *Necator/ Oesophagostomum* spp. and relative abundances of significant bacterial phylum **(A)** and family **(B)** of individual gorillas (natural log-transformed data). LM=linear regression model, SC=Spearman's rank correlation.

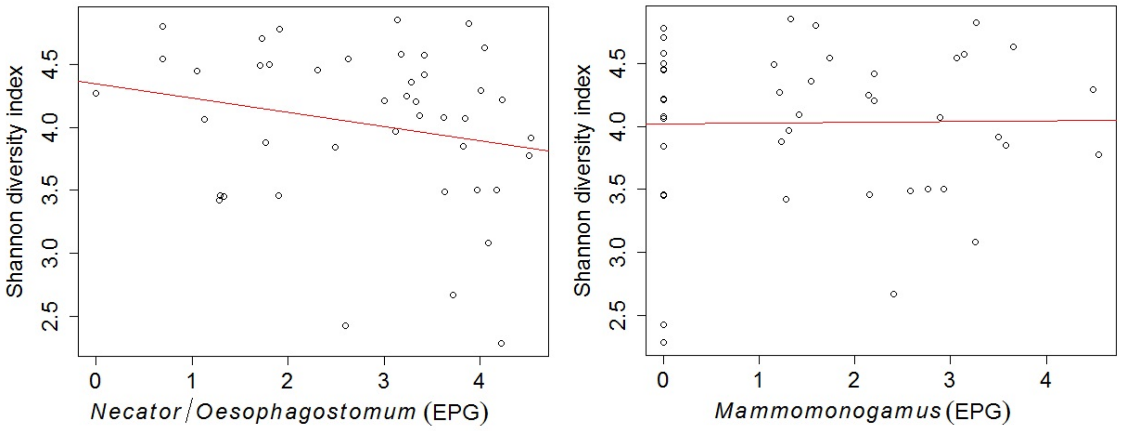

Figure S6. Linear regression model of relationships between GIM diversity (Shannon diversity indices) and egg counts of *Necator/ Oesophagostomum* (p>0.05) and *Mammomonogamus* (p>0.05) (natural log-transformed data).
